# Numerical solution of linear and nonlinear Fredholm integral equations by using weighted mean-value theorem

**DOI:** 10.1186/s40064-016-3645-8

**Published:** 2016-11-14

**Authors:** Ahmet Altürk

**Affiliations:** Department of Mathematics, Amasya University, Ipekköy, Amasya, Turkey

**Keywords:** Linear and nonlinear Fredholm integral equations, Systems of Fredholm integral equations, Systems of Fredholm integro-differential equations, Weighted mean value theorem, Primary 45B05, 35B05, Secondary 45G15

## Abstract

Mean value theorems for both derivatives and integrals are very useful tools in mathematics. They can be used to obtain very important inequalities and to prove basic theorems of mathematical analysis. In this article, a semi-analytical method that is based on weighted mean-value theorem for obtaining solutions for a wide class of Fredholm integral equations of the second kind is introduced. Illustrative examples are provided to show the significant advantage of the proposed method over some existing techniques.

## Introduction and preliminaries

Integral equations have numerous applications in virtually every branches of science. Many physical processes and mathematical models are usually governed by the integral equations. In particular, many initial and boundary value problems can easily be converted to integral equations. Since the subject has many potential application areas, it has attracted many researchers’ attentions from past to today. The literature is very rich of analytical and numerical techniques proposed for solving different kinds of integral equations.

The aim of this article is to propose a simple and effective method for obtaining solutions for a rather wide class of Fredholm integral equations of the second kind. In other words, I investigate linear and nonlinear Fredholm integral and integro-differential equations of the second kind along with the systems of the mentioned classes of Fredholm equations. Before delving into the details of the proposed approach, the list of some available methods proposed by other researchers in the literature are given. The methods with a similar subject area are grouped together, such as wavelet methods (Lepik 2006, 2008; Alpert et al. 1990; Kajani et al. 2006), collocation methods (Zhongying et al. 2006; Maleknejad and Nedaiasl 2011; Jafarian et al. 2013), Adomian decomposition method (Adomian 1994; Wazwaz 1999), transform methods (Ezzati and Mokhtari 2012; Odibat 2008), homotopy perturbation method (Golbabai and Keramati 2008; Abbasbandy 2006), etc. There are also some excellent books from introductory to advanced level, such as Wazwaz (2011), Kress (2014), Rahman (2007) and Pipkin (1991).

The method that is introduced and investigated in this article is weighted integral mean-value method (WMVM). The weighted mean-value theorem are used and applied to the different kinds of Fredholm integral equations. As a result, a linear (or, nonlinear) system of algebraic equations are obtained. By solving these systems of equations, the desired solution for the integral equation will be reached. Elaborated examples are provided to show the applicability and validity of the proposed method.

## Description of the method: weighted mean-value method for integrals (WMVM)

Mean value theorems for both derivatives and integrals are very powerful tools in mathematics. They can be used to obtain very important inequalities and to prove basic theorems of mathematical analysis. Recently, some applications of the mean-value theorem for solving different classes of Fredholm integral equations from one dimensional to higher dimensional have been introduced (Avazzadeh et al. 2011; Heydari et al. 2013; Li and Huang 2016). The results are promising and the method is very simple.

In this article, the weighted mean-value theorem will be used to obtain solutions for a wide class of Fredholm integral equations. As it will be seen in the subsequent sections that under some mild conditions the weighted mean-value theorem can be applied to Fredholm integral equations and significant results are obtained.

### **Theorem 1**

[Weighted mean value theorem for integrals (Apostol 1967)] *Let* $\phi ,\psi {:}\,[a,b] \rightarrow {\mathbb {R}}$ *be continuous on* $[a,\,b]$. *If* $\psi$ *never changes sign in* $[a,\,b]$, *then there exists a number* $c\in [a,b]$ *such that*

$$\begin{aligned} \int _{a}^{b}\phi (x)\psi (x)\,dx = \phi (c)\int _{a}^{b}\psi (x)\,dx. \end{aligned}$$

Results in this paper include application of the weighted mean-value theorem for integrals to the following classes of Fredholm integral equations:

- Linear and nonlinear Fredholm integral equations of the second kind (“Solving linear and nonlinear Fredholm integral equations via WMVM” section)
- Linear and nonlinear Fredholm integro-differential equations of the second kind (“Solving Fredholm integro-differential equations via WMVM” section)
- Linear and nonlinear systems of Fredholm integral equations of the second kind (“Solving linear and nonlinear systems of Fredholm integral equations via WMVM” section)
- Linear and nonlinear systems of Fredholm integro-differential equations of the second kind (“Solving systems of Fredholm integro-differential equations via WMVM” section)

In addition, illustrative examples (see “Numerical results” section) are provided to show the ability of the method and to compare with the existing approaches in the literature (see “Comparison and discussions” section).

I would like to point out that I do not aim for complete generality, but making simplifying assumptions that produce significant results. In Avazzadeh et al. (2011), the authors obtained significant results under the assumption that an application of the mean-value theorem to Fredholm integral equations produces a number *c* rather than a function *c*(*x*). For some cases, this assumption produces an error in numerical solution (Zhong 2013). Throughout the paper I also assume $c(x)=c.$

## Solving linear and nonlinear Fredholm integral equations via WMVM

In this section, consider the following Fredholm integral equation of the second kind:

1 $$\begin{aligned} u(x) = f(x) + \lambda \int _{a}^{b}K(x,t) F(u(t)) \, dt, \quad x,t \in [a,\,b], \end{aligned}$$

where $\lambda$ is a real number, F, f, and K are continuous functions, and *u* is the unknown function to be determined. Since the Eq. (1) will stand for both linear and non-linear Fredholm integral equations, the case that $F(u(\cdot ))=u(\cdot )$ is allowed.

In this and all subsequent sections, the assumption on the kernel function is as follows:

$$\begin{aligned} K(x,t)\ge 0\quad({\mathrm{or}}, K(x,t)\le 0)\,{\mathrm{for\,all}}\,x,t \in [a,\,b]. \end{aligned}$$

After applying WMVM to (1), one gets

2 $$\begin{aligned} u(x) = f(x) + \lambda F(u(c))\gamma (x), \end{aligned}$$

where $\gamma (x)=\int _{a}^{b}K(x,t)\, dt$ and $c\in [a,\,b]$. Notice that to obtain a solution for (1), one just needs to find the value of u(c) for c whose existence guaranteed by weighted mean-value theorem. To reach *u*(*c*) and *c*, the following steps are proposed:

First substitute *c* for *x* in (2) to get

3 $$\begin{aligned} u(c) = f(c) + \lambda F(u(c))\gamma (c). \end{aligned}$$

Then, substitute (2) into (1) to get

4 $$\begin{aligned} u(x) = f(x) + \lambda \int _{a}^{b}K(x,t) F\left( f(t) + \lambda F(u(c))\gamma (t)\right) \, dt. \end{aligned}$$

Next, plug *c* into (4) which lead to

5 $$\begin{aligned} u(c) = f(c) + \lambda \int _{a}^{b}K(c,t) F\left( f(t) + \lambda F(u(c))\gamma (t)\right) \, dt. \end{aligned}$$

After that, solve (3) and (5) simultaneously to obtain *c* and *u*(*c*).

Finally, substitute *c* and *u*(*c*) into (2) to get a solution.

## Solving Fredholm integro-differential equations via WMVM

In this section, consider Fredholm integro-differential equation given by

6 $$\begin{aligned} u^{(n)}(x) = f(x) + \lambda \int _{a}^{b}K(x,t) F(u(t)) \, dt, \quad u^{(k)} = a_k, \quad 0\le k \le n-1, \end{aligned}$$

where $\lambda$, F, f and K are defined as before, $u^{(n)}(x)$ stands for the $n{\text {th}}$ derivative, and $a_k$ are constants that represent the initial conditions.

In operator notation, Eq. (6) can be written as

7 $$\begin{aligned} Lu(x) = f(x) + \lambda \int _{a}^{b}K(x,t) F(u(t)) \, dt, \end{aligned}$$

where the differential operator is given by $L=\frac{d^n}{dx^n}\cdot$ The inverse operator $L^{-1}$ is an *n*-fold integral operator given by

8 $$\begin{aligned} L^{-1}(\cdot ) = \int _{0}^{x} \int _{0}^{x} \ldots \int _{0}^{x} (\cdot )\, dx. \end{aligned}$$

Applying WMVM to (6), one can obtain

9 $$\begin{aligned} u^n(x) = f(x) + \lambda F(u(c))\gamma (x), \end{aligned}$$

where $$\gamma (x)=\int _{a}^{b}K(x,t)\, dt$$ and $$c\in [a,\,b]$$.

An application of the integral operator $L^{-1}$ to both sides of Eq. (9) along with initial conditions yields

10 $$\begin{aligned} u(x) =\sum _{k=0}^{n-1} \frac{a_kx^k}{k!}+L^{-1}\left( f(x)\right) + \lambda L^{-1}\left( F(u(c))\gamma (x)\right) . \end{aligned}$$

Now, replace *x* with *c* in (10) to get

11 $$\begin{aligned} u(c) =\sum _{k=0}^{n-1} \frac{a_kc^k}{k!}+L^{-1}\left( f(c)\right) + \lambda \left( L^{-1}\left( F(u(c))\gamma (x)\right) \Big |_{x=c} \right) . \end{aligned}$$

In addition, substitute Eqs. (9) and (10) into (6) to get

12 $$\begin{aligned} F(u(c))\gamma (x) = \int _{a}^{b}K(x,t) F\left( \sum _{k=0}^{n-1} \frac{a_kt^k}{k!}+L^{-1}\left( f(t)\right) + \lambda L^{-1}\left( F(u(c))\gamma (t)\right) \, dt \right) . \end{aligned}$$

Then, replace *x* with *c* in (12) to get

13 $$\begin{aligned} F(u(c))\gamma (c) = \int _{a}^{b}K(c,t) F\left( \sum _{k=0}^{n-1} \frac{a_kt^k}{k!}+L^{-1}\left( f(t)\right) + \lambda L^{-1}\left( F(u(c))\gamma (t)\right) \, dt \right) . \end{aligned}$$

Finally, considering Eqs. (13) and (11) together, a system of equations with *c* and *u*(*c*) appearing as unknowns are obtained. Solution of this system will give a numerical approximation of desired function *u*(*x*).

## Solving linear and nonlinear systems of Fredholm integral equations via WMVM

In this section, consider systems of Fredholm integral equations given by

14 $$\begin{aligned} u_1(x)& = f_1(x)+\int _{a}^{b}\left( K_{11}(x,t)F_{11}(u_1(t)) + K_{12}(x,t)F_{12}(u_2(t))+\ldots \right) \, dt, \nonumber \\ u_2(x)& = f_2(x)+\int _{a}^{b}\left( K_{21}(x,t)F_{21}(u_1(t)) + K_{22}(x,t)F_{22}(u_2(t))+\ldots \right) \, dt, \nonumber \\&\vdots\end{aligned}$$

It is assumed that there is $n\times n$ system of equations. One particular equation can be represented by

15 $$\begin{aligned} u_i(x) =f_i(x)+\int _{a}^{b}\left( \sum _{j=1}^{n} K_{ij}(x,t)F_{ij}(u_i(t)) \right) \, dt, \quad 1\le i \le n. \end{aligned}$$

If applying WMVM to (15), one gets

16 $$\begin{aligned} \displaystyle u_i(x) =f_i(x)+\sum _{j=1}^{n}F_{ij}\left( u_{j}(c_{j+(i-1)n})\right) \gamma _{j+(i-1)n}(x), \quad 1\le i \le n, \end{aligned}$$

where $\gamma _m(x)=\int _{a}^{b}K_{ij}(x,t)\, dt,\, \, m = j+(i-1)n$ and $c_m\in [a,\,b]$. For simplicity and notational convenience , without loss of generality, it is assumed that there are two unknowns and two functions, i.e., $n=2$.

Thus, one has

17 $$\begin{aligned} u_1(x)& = f_1(x)+\int _{a}^{b}\left( K_{11}(x,t)F_{11}(u_1(t)) + K_{12}(x,t)F_{12}(u_2(t))\right) \, dt, \nonumber \\ u_2(x)& = f_2(x)+\int _{a}^{b}\left( K_{21}(x,t)F_{21}(u_1(t)) + K_{22}(x,t)F_{22}(u_2(t))\right) \, dt. \end{aligned}$$

After applying WMVM to (17), one gets

18 $$\begin{aligned} u_1(x)& = f_1(x)+F_{11}(u_1(c_1))\gamma _1(x)+ F_{12}(u_2(c_2))\gamma _2(x), \nonumber \\ u_2(x)& = f_2(x)+F_{21}(u_1(c_3)) \gamma _3(x)+ F_{22}(u_2(c_4))\gamma _4(x), \end{aligned}$$

where $$c_m\in [a,\, b]$$ and

$$\begin{aligned} \gamma _1(x)& = \int _{a}^{b}K_{11}(x,t)\, dt,\\ \gamma _2(x)& = \int _{a}^{b}K_{12}(x,t)\, dt,\\ \gamma _3(x)& = \int _{a}^{b}K_{11}(x,t)\, dt, \\ \gamma _4(x)& = \int _{a}^{b}K_{12}(x,t)\, dt. \end{aligned}$$

Substituting $c_1$ and $c_3$ into first equation in (18) and $c_2$ and $c_4$ into second equation in (18) yields

19 $$\begin{aligned} u_1(c_1)& = f_1(c_1)+F_{11}(u_1(c_1))\gamma _1(c_1)+ F_{12}(u_2(c_2))\gamma _2(c_1), \nonumber \\ u_1(c_3)& = f_1(c_3)+F_{11}(u_1(c_1))\gamma _1(c_3)+ F_{12}(u_2(c_2))\gamma _2(c_3) ,\nonumber \\ u_2(c_2)& = f_2(c_2)+F_{21}(u_1(c_3)) \gamma _3(c_2)+F_{22}(u_2(c_4))\gamma _4(c_2), \nonumber \\ u_2(c_4)& = f_2(c_4)+F_{21}(u_1(c_3)) \gamma _3(c_4)+ F_{22}(u_2(c_4))\gamma _4(c_4). \end{aligned}$$

4 more equations are needed in order to have 8 equations with 8 unknowns. To obtain other 4 equations, substitute (18) into (17) and get

20 $$\begin{aligned} u_1(x)& = f_1(x)+\int _{a}^{b}\left( K_{11}(x,t)F_{11}\left( f_1(t)+F_{11}(u_1(c_1))\gamma _1(t)+F_{12}(u_2(c_2))\gamma _2(t)\right) \right. \nonumber \\&\quad \left. +\,K_{12}(x,t)F_{12}\left( f_2(t)+F_{21}(u_1(c_3)) \gamma _3(t)+ F_{22}(u_2(c_4))\gamma _4(t)\right) \right) \, dt, \end{aligned}$$

and

21 $$\begin{aligned} u_2(x)& = f_2(x)+\int _{a}^{b}( K_{21}(x,t)F_{21}\left( f_1(t)+F_{11}(u_1(c_1))\gamma _1(t)+ F_{12}(u_2(c_2))\gamma _2(t)\right) \nonumber \\&\quad +K_{22}(x,t)F_{22}\left( f_2(t)+F_{21}(u_1(c_3)) \gamma _3(t)+ F_{22}(u_2(c_4))\gamma _4(t)\right) )\, dt. \end{aligned}$$

Replacing *x* with $c_1$ and $c_3$ in (20), and $c_2$ and $c_4$ in (21) one can get 4 more equations. Combining these equations with (19), a system of algebraic equations will be obtained. By solving this algebraic system of equations, the desired solution for the system of integral equations will be reached.

## Solving systems of Fredholm integro-differential equations via WMVM

In this sections, systems of Fredholm integro-differential equations of the second kind will be studied. Consider

22 $$\begin{aligned} u_1^{(n)}(x)& = f_1(x)+\int _{a}^{b}\left( K_{11}(x,t)F_{11}(u_1(t)) + K_{12}(x,t)F_{12}(u_2(t))\right) \, dt, \quad u_1^{(k)} = a_k, \quad 0\,\le\,k\,\le\,n-1,\nonumber \\ u_2^{(n)}(x)& = f_2(x)+\int _{a}^{b}\left( K_{21}(x,t)F_{21}(u_1(t)) + K_{22}(x,t)F_{22}(u_2(t))\right) \, dt, \quad u_2^{(k)} = b_k, \quad 0\,\le\,k\,\le\,n-1. \end{aligned}$$

After applying WMVM to (22), one gets

23 $$\begin{aligned} u_1^{(n)}(x)& = f_1(x)+F_{11}(u_1(c_1))\gamma _1(x)+ F_{12}{u_2(c_2)}\gamma _2(x), \nonumber \\ u_2^{(n)}(x)& = f_2(x)+F_{21}(u_1(c_3)) \gamma _3(x)+ F_{22}{u_2(c_4)}\gamma _4(x), \end{aligned}$$

where $$c_m\in (a,\,b)$$ and

$$\begin{aligned} \gamma _1(x)=\int _{a}^{b}K_{11}(x,t)\, dt, \,\,\, \gamma _2(x)=\int _{a}^{b}K_{12}(x,t)\, dt, \,\,\,\gamma _3(x)=\int _{a}^{b}K_{11}(x,t)\, dt, \,\,\, \gamma _4(x)=\int _{a}^{b}K_{12}(x,t)\, dt. \end{aligned}$$

An application of the integral operator $L^{-1}$ introduced in (8) to both sides of Eq. (23) along with initial conditions yields

24 $$\begin{aligned} u_1(x)& = \sum _{k=0}^{n-1} \frac{a_kx^k}{k!}+L^{-1}\left( f(x)\right) + L^{-1}\left( F_{11}(u_1(c_1))\gamma _1(x)+ F_{12}{u_2(c_2)}\gamma _2(x)\right) ,\nonumber \\ u_2(x)& = \sum _{k=0}^{n-1} \frac{b_kx^k}{k!}+L^{-1}\left( f(x)\right) + L^{-1}\left( F_{21}(u_1(c_3)) \gamma _3(x)+ F_{22}{u_2(c_4)}\gamma _4(x) \right) . \end{aligned}$$

Substituting $c_1$ and $c_3$ into first equation in (24) and $c_2$ and $c_4$ into second equation in (24), 4 equations will be obtained. Then, by substituting (23) and (24) into (22), 2 new equations will be obtained. Replacing *x* with $c_1$ and $c_3$ in the first equation and $c_2$ and $c_4$ in the second equation, there will be 4 more equations. Solving this nonlinear system of equations will give the desired solution.

## Numerical results

In this section, numerical results are presented for various types of Fredholm integral equations mentioned in the previous sections. The results show the validity and efficiency of the method. It is important to note that all numerical computations are performed using Matlab software. For solving a non-linear system of equations, the Matlab built-in functions use the Newton’s method with an initial guess or some modified versions of it. Since these methods are, in general, local, the initial guess plays a decisive role in obtaining solutions.

### *Example 1*

(*Linear Fredholm integral equation*) Consider the following linear Fredholm integral equation of the first kind (Wazwaz 2011):

$$\begin{aligned} u(x)= e^{x+2}-2 \int _{0}^{1} e^{x+t}u(t)\,dt. \end{aligned}$$

The exact solution for the equation is that $u(x)=e^x$.

Applying the presented method, the following system of equations are obtained:

25 $$\begin{aligned} u(c)& = e^{c+2}-2(e-1)e^cu(c), \nonumber \\ u(c)& = e^{c+2}(2-e^2)+2(e-1)^2(e+1)u(c)e^c. \end{aligned}$$

Solving this system of nonlinear equations results in

$$\begin{aligned} c = 0.620114506958278 \,\,\,\text{ and } \,\,\,u(c) =1.859140914229523. \end{aligned}$$

**Fig. 1:**
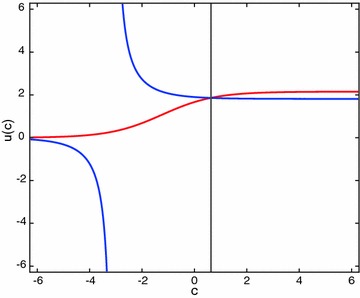
Graphs of the equations given in (25). The intersection point c agrees with the solution obtained above

The approximate solution can be evaluated from

$$\begin{aligned} u_{ap}(x)=e^{x+2}-2(e-1)e^xu(c), \end{aligned}$$

which leads to the exact solution. The graph of the equations in (25) is given in Fig. 1.

### *Example 2*

(*Linear Fredholm integral equation*) Consider the following linear Fredholm integral equation of the first kind (Mikaeilvand and Noeiaghdam 2014):

$$\begin{aligned} u(x)= x^3-2(3+\cos (1)-4\sin (1))(\cos (x)+\sin (x))+ \int _{0}^{1} [\sin (x+t)+\cos (x+t)]u(t)\,dt. \end{aligned}$$

The exact solution for the equation is that $u(x)=x^3$.

Applying the presented method, the following system of equations are obtained:

$$\begin{aligned} u(c)& = c^3 + (\cos (c) + \sin (c))\left[ u(c)(1+\sin (1)-\cos (1))-2(\cos (1)-4\sin (1))\right] , \\ u(c)& = c^3 + (\cos (c) + \sin (c))\left[ (\cos (2)-1)(3-4\sin (1)+\cos (1))-2(3+\cos (1)-4\sin (1))\right. \\&\left. \quad -\,\frac{1}{2}u(c)(1+\sin (1)-\cos (1))(cos(2)-3)\right] . \end{aligned}$$

Solving this system of nonlinear equations with the initial guess $[0.5,\,0.5]$ results in

$$\begin{aligned} c = 0.6448066930020793 \,\,\,\text{ and } \,\,\,u(c) =0.2680949356676439. \end{aligned}$$

The approximate solution becomes

$$\begin{aligned} u_{ap}(x)=x^3-1.6653\times 10^{-16}(\sin (x)+cos(x)). \end{aligned}$$

### *Example 3*

(*Nonlinear Fredholm integral equation*) Consider the following nonlinear Fredholm integral equation of the second kind (Wazwaz 2011):

$$\begin{aligned} u(x)= \frac{5}{6}x+ \int _{0}^{1} xt^2u^3(t)\,dt, \quad x,t \in [0,\,1]. \end{aligned}$$

Three exact solutions for the equation are

26 $$\begin{aligned} u(x)=x,\, \frac{\left( \sqrt{21}-1\right) x}{2},\, \text{ and } -\frac{\left( \sqrt{21}+1\right) x}{2}. \end{aligned}$$

Applying the presented method, the following system of equations are obtained:

27 $$\begin{aligned} u(c)& = c\left( \frac{5+2u^3(c)}{6}\right) , \nonumber \\ u(c)& = c\left( \frac{5}{6}+\frac{1}{6}\left( \frac{5+2u^3(c)}{6}\right) ^3\right) . \end{aligned}$$

Solving this system of nonlinear equations yields

28 $$\begin{aligned} c& = 0.793700526076704 \,\,\,\text{ and } \,\,\,u(c) = \,\,0.793700525984100,\nonumber \\ c& = 0.793700526076704 \,\,\,\text{ and } \,\,\,u(c) = \,\,1.421746106732151,\nonumber \\ c& = 0.793700526076704 \,\,\,\text{ and } \,\,\,u(c) =-2.215446632716251. \end{aligned}$$

The approximate solution can be calculated from

$$\begin{aligned} u_{ap}(x)=x\left( \frac{5+2u^3(c)}{6}\right) . \end{aligned}$$

It is important to point out that each pair of solutions given in (28) corresponds to one exact solution given in (30). The first pair leads to the exact solution $u(x)=x$, the second pair leads to the exact solution $u(x)=\frac{(\sqrt{21}-1)x}{2}$, and the last pair leads to the third exact solution $u(x)=-\frac{(\sqrt{21}+1)x}{2}$.

**Fig. 2:**
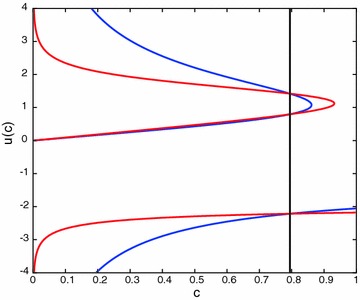
Graphs of the equations given in (27). The intersection points agree with the numerical solutions above

The graph of the equations in (27) is given in Fig. 2.

### *Example 4*

(*Fredholm integro-differential equation*) Consider the following Fredholm integro-differential equation (Rahman 2007):

$$\begin{aligned} u''(x)= e^x - x + \int _{0}^{1}xtu(t)\,dt, \quad u(0)=u'(0) =1, \quad x,t \in [0,\,1]. \end{aligned}$$

The exact solution for the equation is that $u(x)=e^x$.

Applying the presented method, the following system of equations are obtained:

29 $$\begin{aligned} u(c)& = e^c+\left( \frac{u(c)-2}{12}\right) c^3, \nonumber \\ cu(c)& = 2c+\frac{cu(c)-2c}{30}. \end{aligned}$$

Solving this nonlinear system, one gets

30 $$\begin{aligned} c& = 0 \quad \text{ and } \quad u(c) =1,\nonumber \\ c& = \log (2) \quad \text{ and }\quad u(c) =2. \end{aligned}$$

The approximate solution can be calculated from

31 $$\begin{aligned} u_{ap}(x)=e^x+\left( \frac{u(c)-2}{12}\right) x^3. \end{aligned}$$

Substituting that $u(c)=2$ into (31) results in

$$\begin{aligned} u_{ap}(x)=e^x, \end{aligned}$$

which is indeed the exact solution.

### *Example 5*

(*System of Fredholm integral equation*) Consider the following nonlinear system of Fredholm integral equation (Babolian et al. 2004):

32 $$\begin{aligned} u_1(x)& = x-\frac{5}{18}+\int _{0}^{1}\frac{1}{3}\left( u_1(t) + u_2(t)\right) \, dt, \nonumber \\ u_2(x)& = x^2-\frac{2}{9}+\int _{0}^{1}\frac{1}{3}\left( u_1^2(t) + u_2(t)\right) \, dt. \end{aligned}$$

The exact solution for the equation is that $u_1(x)=x$ and $u_2(x)=x^2$.

Applying the presented method, the following system of equations are obtained:

33 $$\begin{aligned} u_1(x)& = x-\frac{5}{18}+\frac{1}{3}\left( u_1(c_1) + u_2(c_2)\right) , \nonumber \\ u_2(x)& = x^2-\frac{2}{9}+\frac{1}{3}\left( u_1^2(c_3) + u_2(c_4)\right) , \end{aligned}$$

where $$c_1, c_2, c_3,$$ and $$c_4\in [0,1]$$.

First substitute $c_1$ and $c_3$ into the first equation in (33), and $c_2$ and $c_4$ into the second equation in (33) to get

34 $$\begin{aligned} u_1(c_1)& = c_1-\frac{5}{18}+\frac{1}{3}\left( u_1(c_1) + u_2(c_2)\right) , \nonumber \\ u_1(c_3)& = c_3-\frac{5}{18}+\frac{1}{3}\left( u_1(c_1) + u_2(c_2)\right) , \nonumber \\ u_2(c_2)& = c_2^2-\frac{2}{9}+\frac{1}{3}\left( u_1^2(c_3) + u_2(c_4)\right) ,\nonumber \\ u_2(c_4)& = c_4^2-\frac{2}{9}+\frac{1}{3}\left( u_1^2(c_3) + u_2(c_4)\right) . \end{aligned}$$

Then plug (33) into (32) to get

35 $$\begin{aligned} u_1(x)& = x-\frac{5}{18}+\frac{1}{9}\left( u_1(c_1) + u_2(c_2)+u_1^2(c_3) + u_2(c_4)+1 \right) ,\nonumber \\ u_2(x)& = x^2-\frac{2}{9}+\frac{1}{3}\left( \frac{(u_1(c_1)+u_2(c_2))^2}{9}+\frac{4(u_1(c_1)+u_2(c_2))}{27}+\frac{u_1^2(c_3) + u_2(c_4)}{3}+\frac{79}{324}\right) . \end{aligned}$$

Now replace *x* with $c_1$ and $c_3$ in the first equation in (35) and $c_2$ and $c_4$ in the second equation in (35) so that there are 4 more equations. Combining (34) with these equations, one finally gets a nonlinear system of 8 equations with 8 unknowns. Solving this system and the result is as follows:

$$\begin{aligned} u_1(c_1)& = c_1= \frac{5}{6} \quad u_1(c_3) =c_3 = \frac{\sqrt{6}}{3}, \\ u_2(c_2)& = u_2(c_4) = c_2=c_4=0.\\ \end{aligned}$$

Substitute these values into (33), the exact solutions are obtained, namely,

36 $$\begin{aligned} u_1(x)=x \quad \text{ and }\quad u_2(x)=x^2. \end{aligned}$$

### *Example 6*

(*System of Fredholm integro-differential equation*) Consider the following system of Fredholm integro-differential equation:

37 $$\begin{aligned} u'_1(x)& = 1-\frac{5}{6}x+\int _{0}^{1}x\left( u_1(t) + u_2(t)\right) \, dt,\quad u_1(0)=0, \nonumber \\ u'_2(x)& = 2x-\frac{1}{12}+\int _{0}^{1}t\left( u_1(t) - u_2(t)\right) \,dt,\quad u_2(0)=0. \end{aligned}$$

The exact solution for the equation is that $u_1(x)=x$ and $u_2(x)=x^2$.

Applying the presented method, the following system of equations are obtained:

38 $$\begin{aligned} u'_1(x)& = 1-\frac{5}{6}x+\left( u_1(c_1) + u_2(c_2)\right) x, \nonumber \\ u'_2(x)& = 2x-\frac{1}{12}+\frac{1}{2}\left( u_1(c_3) - u_2(c_4)\right) , \end{aligned}$$

where $$c_1, c_2, c_3,$$ and $$c_4\in [0,1]$$.

An application of the integral operator $L^{-1}$ introduced in (8) to both sides of Eq. (38) along with initial conditions yields

39 $$\begin{aligned} u_1(x)& = x-\frac{5}{12}x^2+\frac{1}{2}x^2 \left( u_1(c_1) + u_2(c_2)\right) , \nonumber \\ u_2(x)& = x^2-\frac{1}{12}x+\frac{1}{2}\left( u_1(c_3) - u_2(c_4)\right) x. \end{aligned}$$

First substitute $c_1$ and $c_3$ into the first equation in (39), and $c_2$ and $c_4$ into the second equation in (39) to get

40 $$\begin{aligned} u_1(c_1)& = c_1-\frac{5}{12}c_1^2+\frac{1}{2}c_1^2\left( u_1(c_1) + u_2(c_2)\right) , \nonumber \\ u_1(c_3)& = c_3-\frac{5}{12}c_3^2+\frac{1}{2}c_3^2\left( u_1(c_1) + u_2(c_2)\right) , \nonumber \\ u_2(c_2)& = c_2^2-\frac{1}{12}c_2+\frac{1}{2}c_2\left( u_1(c_3) - u_2(c_4)\right) ,\nonumber \\ u_2(c_4)& = c_4^2-\frac{1}{12}c_4+\frac{1}{2}c_4\left( u_1(c_3) - u_2(c_4)\right) . \end{aligned}$$

Then plug (39) and (38) into (37) to get

41 $$\begin{aligned} (u_1(c_1)+u_2(c_2))x& = \frac{1}{72}\left( 12(u_1(c_1)+u_2(c_2))+ 18(u_1(c_3) - u_2(c_4))+47\right) x,\nonumber \\ u_1(c_3) - u_2(c_4)& = \frac{3}{16}( u_1(c_1) + u_2(c_2))+\frac{1}{96}. \end{aligned}$$

Now replace *x* with $c_1, c_2$ and $c_4$ in the first equation in (41) and taking the second equation in (41) as it is there will be 4 more equations. Combining (40) with these equations to get a nonlinear system of 8 equations with 8 unknowns. Solving this system and the result is as follows:

$$\begin{aligned} u_1(c_1)& = c_1= \frac{5}{6} \quad u_1(c_3) =c_3 = \frac{1}{6}, \\ u_2(c_2)& = u_2(c_4) = c_2=c_4=0.\\ \end{aligned}$$

Substitute these values into (39), the exact solutions are obtained, namely,

42 $$\begin{aligned} u_1(x)=x \quad \text{ and }\quad u_2(x)=x^2. \end{aligned}$$

## Comparison and discussions

In this section, the results obtained in this article and those obtained by applying some well-known methods will be compared. In particular, I will be interested in comparison with the Adomian decomposition method (ADM). It was introduced in Adomian (1994). The ADM is a breakthrough achievement in differential and integral equations. Since then, the method is applied to various differential equations, integral equations, and even partial differential equations. Let me first briefly mention about the ADM. The decomposition method threats the unknown function differently in the sense that if the unknown function appears linearly in an integral equation, the representation becomes a series representation whose terms considered as components of the unknown function, i.e.,

$$\begin{aligned} u(x)=\sum _{n=0}^{\infty }u_{k}(x), \end{aligned}$$

and if it appears nonlinearly in an integral equation, i.e., *F*(*u*(*x*)), the representation admits a series of so-called Adomian polynomials $A_n$ given by

43 $$\begin{aligned} A_{n}=\frac{1}{n!}\frac{d^n}{d\lambda ^n}\left[ F\left( \sum _{k=0}^{n} \lambda ^ku_{k}\right) \right] _{\lambda =0}, \quad n=0, 1, 2, \ldots . \end{aligned}$$

For a detailed treatment of application of the ADM to integral equations the reader is referred to Wazwaz (2011).

### *Example 7*

(*Nonlinear Fredholm integral equation*) Consider the following nonlinear Fredholm integral equation of the second kind:

$$\begin{aligned} u(x)= \frac{5}{6}x+ \int _{0}^{1} xt^2u^3(t)\,dt, \quad x \in [0,\,1]. \end{aligned}$$

This was the second example in the previous section. Applying the ADM, one gets

$$\begin{aligned} \sum _{n=0}^{\infty }u_n(x)= \frac{5}{6}x+ \int _{0}^{1} xt^2\left( \sum _{n=0}^{\infty }A_n(t)\right) \,dt, \end{aligned}$$

where $A_n$ are the Adomian polynomials given in (43).

The ADM admits the following recursion relation:

$$\begin{aligned} u_0(x)& = \frac{5}{6}x,\nonumber \\ u_{k+1}(x)& = \int _{0}^{1} xt^2A_k(t)\,dt, \quad k\ge 0. \end{aligned}$$

This yields

$$\begin{aligned} u_0(x)& = \frac{5}{6}x,\\ u_1(x)& = \int _{0}^{1} xt^2A_0(t)\,dt = \frac{125}{1296}x,\\ u_2(x)& = \int _{0}^{1} xt^2A_1(t)\,dt = \frac{3125}{93312}x,\\&\vdots&\end{aligned}$$

Combining these components of the solutions to get

$$\begin{aligned} u(x)=\left( \frac{5}{6}x + \frac{125}{1296}x + \frac{3125}{93312}x + \ldots \right) \approx x. \end{aligned}$$

It is important to note here that an application of the ADM produced one approximate solution. On the other hand, applying the WMWM (see Example 2) 3 exact solutions were obtained.

As the final example, consider an equation for which the method introduced in Avazzadeh et al. (2011) does not provide a number $c\in [0,1]$ when solving the nonlinear system of equations obtained after applying the method. This is shown by a geometric reasoning. it is also shown that applying WMVM will produce the exact solution.

### *Example 8*

Consider the following linear Fredholm integral equation of the first kind:

$$\begin{aligned} u(x)= -x^2+x+\frac{2}{3}+\int _{0}^{1}(x-t)^2u(t)\,dt. \end{aligned}$$

The exact solution for the equation is that $u(x)=1$.

Applying the method introduced in Avazzadeh et al. (2011), the following system of equations are obtained:

44 $$\begin{aligned} u(c)& = -c^2+c+\frac{2}{3}, \nonumber \\ u(c)& = \frac{5}{4}\left( c^2(2-2c+c^2)u(c)+\frac{30c(1-c)+169}{180} \right) . \end{aligned}$$

**Fig. 3:**
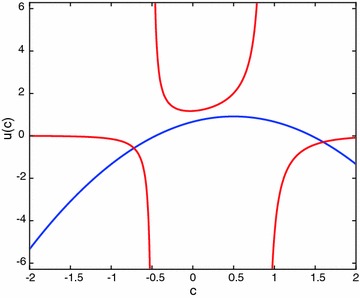
Graphs of the equations given in (44). No intersection point between 0 and 1

The graph of the equations in (44) is given in Fig. 3.

From the Fig. 3, it is clear that one cannot find a number *c* between 0 and 1 satisfying both equations given in (44). On the other hand, applying the presented method, one gets

45 $$\begin{aligned} u(x)& = -x^2+x+\frac{2}{3}+u(c)\int _{0}^{1}(x-t)^2\,dt\nonumber \\& = -x^2+x+\frac{2}{3}+u(c)\left( x^2-x+\frac{1}{3}\right) . \end{aligned}$$

Substitute *c* for *x* to get

46 $$\begin{aligned} u(c)=1. \end{aligned}$$

Using (46), the second equation [see (4)] directly gives the exact solution. That is, $u(x)=1.$

## Conclusion

In this article, an effective method based on weighted mean-value theorem for solving different types of Fredholm integral equations of the second kind, from linear to nonlinear equations and integro-differential to the systems of equations involving them, is presented. The numerical and analytical solutions are conducted using Matlab. Thoroughly worked-out examples are provided in order to show the accuracy and applicability of the presented approach.
